# Roux-en-Y gastric bypass-induced perturbative changes in microbial communities and metabolic pathways in rats

**DOI:** 10.3389/fmicb.2022.1034839

**Published:** 2022-11-10

**Authors:** Jing Yang, Lei Chen, Xue-Ying Shang, Yi-Lin Chen, Shan-Shan Zhao, Shi Jin, Jing Yang, Hui-Xin Liu, Jian Du

**Affiliations:** ^1^Department of Endocrinology, The Fourth Affiliated Hospital of China Medical University, Shenyang, China; ^2^Liaoning Key Laboratory of Obesity and Glucose/Lipid Associated Metabolic Diseases, China Medical University, Shenyang, China; ^3^Health Sciences Institute, China Medical University, Shenyang, China; ^4^Institute of Life Sciences, China Medical University, Shenyang, China

**Keywords:** gut microbiota, obesity, RYGB, SG, metabolism remodeling

## Abstract

**Background:**

Obesity has become a global health and socioeconomic problem because of an inadequate balance between energy intake and energy expenditure. Roux-en-Y gastric bypass (RYGB) and sleeve gastrectomy (SG) are the two most commonly used strategies for weight loss, which have been proven to benefit from gut microbiota restoration.

**Methods:**

Rats received SG, RYGB, and sham operations for 10 weeks. At the end of the experiment, the fecal microbiota was analyzed using 16s rRNA gene sequencing. In addition, the shift in the plasma metabolism of rats that underwent RYGB surgery was analyzed using untargeted metabolomics. The crosstalk between microbiome and metabolites was revealed using metabolic pathway enrichment and integrated analysis.

**Result:**

The SG surgery induced a modest shift in the gut microbiota relative to the RYGB. RYGB significantly decreased the alpha diversity and *Firmicutes*/*Bacteroides* (F/B) ratio and increased the proportion of *Escherichia, Bacteroides*, and *Akkermansia* genera compared to sham and SG operations. The predicted function of gut microbiota revealed that the RYGB surgery uniquely enhanced the capability of linoleic acid and sphingolipid metabolism. Furthermore, the circulating serine, phosphatidylcholine (PC) 20:5/22:5, riboflavin, L–carnitine, and linoleic acid were evaluated after RYGB surgery. In addition, the metabolic pathway enrichment and integrated analysis suggest that the RYGB induced *Escherichia, Bacteroides*, and *Akkermansia* might inhibit the sphingonine and phytosphingosine metabolisms from serine and promote the PC (20:5/22:5) metabolism to produce linoleic acid.

**Conclusion:**

This comprehensive analysis not only revealed the difference in the gut microbiota shifts after SG and RYGB but also discovered the perturbative changes in microbial communities and metabolic pathways after RYGB surgery, which provided clues for improving the beneficial effect of RYGB in metabolic disease intervention *via* regulating bacterial-metabolite crosstalk.

## Introduction

Obesity has become a global health and socioeconomic problem (NCD Risk Factor Collaboration, 2017). The inadequate balance between energy intake and energy expenditure contributes to obesity and metabolic diseases (de Clercq et al., 2016). An increasing number of evidence revealed the involvement of gut microbiota in obesity development. A higher phylum *Firmicutes*/*Bacteroides* (F/B) ratio promotes intestinal caloric intake and contributes to fat accumulation. In addition, restoring the dysbiosis of gut microbiota has become a candidate strategy for obesity intervention.

Roux-en-Y gastric bypass (RYGB) and sleeve gastrectomy (SG) are the two most effective strategies for weight loss (Dang et al., 2022). The patients who received surgery showed a decreased fat mass and caloric intake, as well as nutrient absorption and insulin resistance (Miras and le Roux, 2013). RYGB surgery could increase serum glucose excretion and enteroendocrine cell glucagon-like peptide (GLP)-1 release from the small intestine (Wallenius et al., 2020; Kwon et al., 2021). Meanwhile, RYGB promotes the development of nephrolith *via* increasing oxalic acid (Lieske, 2017). In addition, RYGB and SG could also induce adverse outcomes such as significant bone loss (Scibora et al., 2015). It has been well-documented that the dysbiosis of the gut microbiota contributes to kidney stone formation and bone loss (Ticinesi et al., 2018; Ibanez et al., 2019). The benefits of RYGB and SG surgeries have been linked to the shift in gut microbiota and fecal metabolites (Li J. V. et al., 2021; Dang et al., 2022). RYGB induced more profound metabolic effects *via* increasing the proportion of *Actinobacteria* and *Proteobacteria* phyla but decreasing the lumen-conjugated and secondary bile acids (Haange et al., 2020; Zhang et al., 2022). In addition, the increased pro-inflammatory bacterial species such as lipopolysaccharide (LPS) and flagellin were also observed in the feces of patients who received RYGB (Scheithauer et al., 2022). Gut microbiota-mediated metabolite lithocholic acid (LCA), which increased after SG surgery, could activate the vitamin D receptor to ameliorate diabetic phenotypes (Hu et al., 2015; Chaudhari et al., 2021). Taken together, the gut microbiota plays an important role in the outcomes of RYGB and SG. However, systemic evaluation of RYGB and SG in restoring gut microbiota and circulating metabolites has been rarely studied.

In the present study, rats received RYGB and SG surgeries. The fecal microbiota was analyzed using 16s rRNA gene sequencing, and the shift in plasma metabolism after RYGB surgery was analyzed using untargeted metabolomics. Our comprehensive analysis contributes to a better understanding of the profound bacterial-metabolite crosstalk after RYGB surgery and provides a possible strategy for enhancing therapies and decreasing adverse outcomes by restoring gut microbiota and related metabolites.

## Materials and methods

### Animals and bariatric surgical protocol

Male SD rats that were 8 weeks old were housed in a specific pathogen-free environment at 24–26°C with a 12-h light-dark cycle. All animal procedures used in this study were conducted according to the Guide for Care and Use of Laboratory Animals and approved by the Animal Ethics Committee of China Medical University (No. 2019099). All efforts have been made to minimize the suffering of the animals. The rats were divided into four groups with six rats per group as follows: SG sham (SGS), SG, RYGB sham (RYGBS), and RYGB after 1 week of adaptation. SG, RYGB, and corresponding sham operations were performed as previously described (Shang et al., 2021). After 10 weeks, the animals were anesthetized and sacrificed. Blood and feces were collected for further analysis.

### Fecal microbiota 16S rRNA gene sequencing

Fecal DNA was isolated, and the V3+V4 hypervariable regions of the bacterial 16S rRNA gene were amplified and sequenced on the Illumina MiSeq PE300 platform. The PCR conditions used to amplify the prokaryotic 16S fragments consisted of an initial denaturation at 98 C for 30 s; 35 cycles of denaturation at 98 C for 10 s, annealing at 54 C/52 C for 30 s, and extension at 72 C for 45 s; and a final extension at 72 C for 10 min. QIIME Version 2.0 was used for analyzing raw sequencing reads (Caporaso et al., 2010). Uparse software (Version 7.1) was used to cluster the same operational taxonomic units (OTUs) with ≥97% similarity sequences. The Greengenes 16S rRNA gene reference database was adopted to classify OTU taxonomically.

### Analysis of flora microbiota diversity, structure, and predictive function

The OTU numbers of each sample were flattening, and the alpha diversity of fecal bacteria was calculated based on the normalized OTU table using the R package Vegan as described in previous methods (Sheng et al., 2017; Wang et al., 2018). Principal coordinate analysis (PCoA) and similarities (PERMANOVA) were used to reveal the difference in stool microbiome profile based on the OTU level and were analyzed using the R package Vegan. Besides, a sequence of OTUs represented in samples was used to predict the function of the intestinal microbiome by PICRUST2 as previously described (Liu et al., 2016), and significantly changed KEGG pathways were tested by the two-way ANOVA test and Tukey's multiple-comparisons test.

### Analysis of plasma metabolomics

Blood metabolite signatures were identified by liquid chromatography-mass spectrometry (LC-MS) between RYGBS and RYGB groups' samples. The processed data, such as m/z, RT, and normalized peak area percentages, were imported into SIMCA to identify metabolites. The HMDB database was adopted to map and identify the metabolites. Partial least squares-discriminant analysis (PLS-DA) was used to reveal the metabolite changes in groups, such as R package ropls, and the abundance of significant metabolites with variable important in projection (VIP) ≥1 and *p*-value (Wilcoxon test) < 0.05 were selected for enrichment analysis. The enrichment pathway of the differential plasma metabolite profile between the two groups was analyzed using MetaboAnalyst 5.0 (http://www.metaboanalyst.ca).

### Correlation and co-occurrence network analysis

Spearman's correlation of the significant KEGG pathways of bacterial predicted function and remarkably changed bacteria, alpha diversity of microbiota, and microbiome as well as blood metabolites and gut bacteria was performed using R package dplyr. Meanwhile, the co-occurrence network of metabolites of key pathways and changed bacterial metabolites was constructed and visualized using R package igraph and Cytoscape, respectively.

### Statistical analysis

For statistics in multiple groups, we utilized the Kruskal-Wallis and two-way ANOVA tests to evaluate the difference among groups. *Post-hoc* Dunn's test, Tukey's multiple-comparisons test, and the Wilcoxon test were performed to analyze the difference between the two groups. Values of *P* < 0.05 were considered statistically significant. The error bars indicate the mean ± standard error of the mean (SEM).

## Results

### SG and RYGB induced gut microbiota alteration in rats based on the 16S rRNA gene

**Figure 1:**
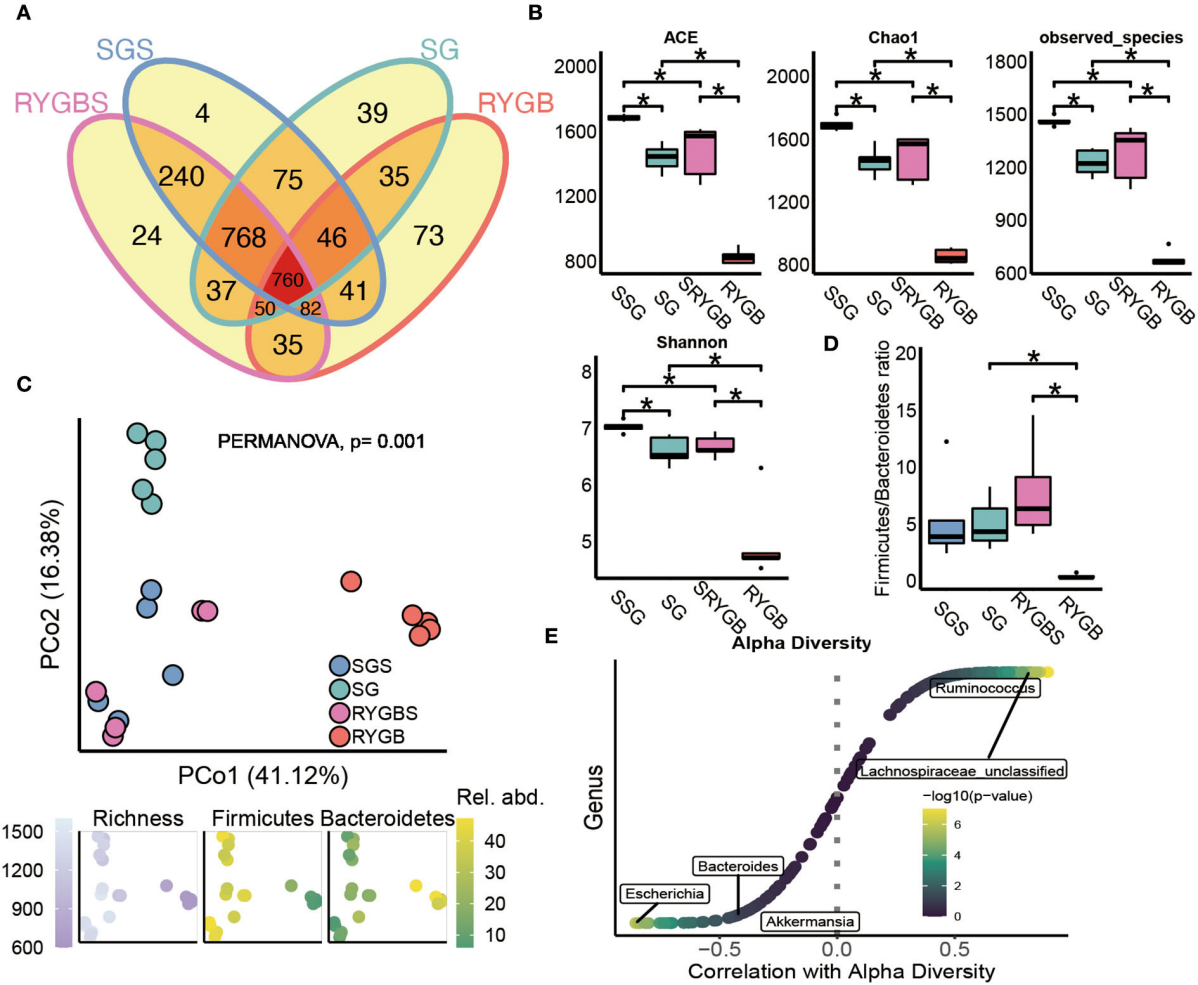
The altered fecal microbiota of SG and RYGB surgeries. **(A)** The shared and unique observed OTUs in four groups. **(B)** The alpha diversity difference among groups. **(C)** PCoA reveals a clear distinct difference in gut microbiota in four groups. **(D)** The comparison of phylum *Firmicutes*/*Bacteroidetes* ratio in four group individuals. **(E)** The correlation between alpha diversity and alerted gut microbiome. *P < 0.05, Kruskal–Wallis test with *post-hoc* Dunn's test.

As shown in Figure 1A, the shared OTUs between RYGB and RYGBS (927) were lower than that between SG and SGS (1,649). The ACE, chao1, observed species, and Shannon index in SG and RYGB groups significantly decreased compared to that in the SGS and RYGBS groups, respectively (Figure 1B). The PCoA analysis revealed clear but distinct discrimination in four groups, and RYGB induced more gut microbiota structure alteration than SG, which was indicated by the richness index of the F/B ratio (Figure 1C). Consistently, the F/B ratio of RYGB was significantly decreased compared to RYGBS and SG (Figure 1D). Moreover, *Ruminococcus* and *Lachnospiraceae_unclassified* genera were positively correlated to alpha diversity (Figure 1E). In addition, the negative association between alpha diversity and *Escherichia, Bacteroides*, and *Akkermansia* genera was also revealed by Spearman's correlation analysis (Figure 1E).

### The specific change in gut microbiota after SG and RYGB in rats

**Figure 2:**
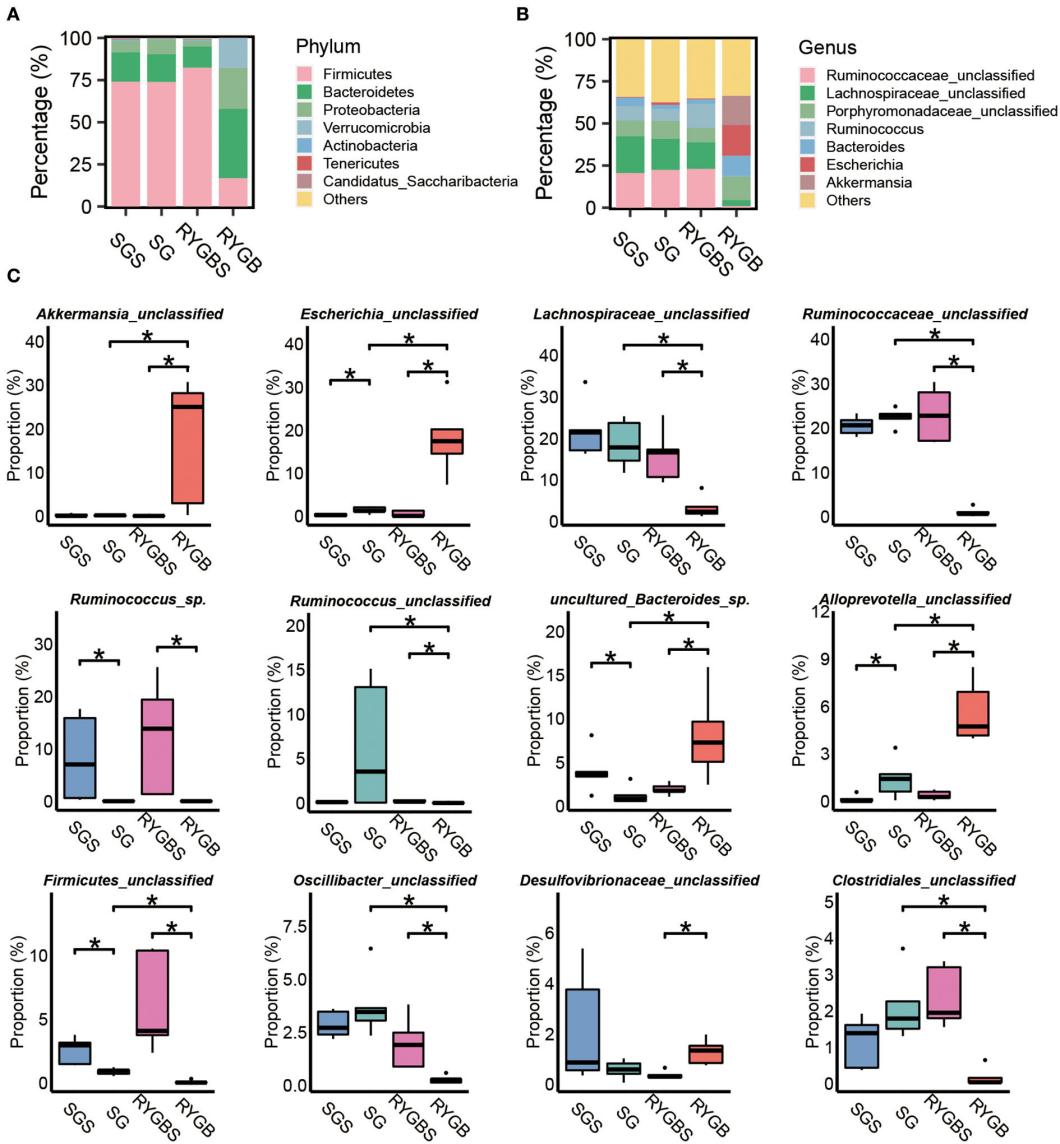
Identification of specific altered bacteria for SG and RYGB. **(A,B)** Stacked bar plots depicting the percentage of phylum (left) and genus (right) of intestinal bacteria in the screening results. **(C)** Significantly changed bacteria at the genus levels in gut microbiota. *P < 0.05, Kruskal–Wallis test with *post-hoc* Dunn's test.

Next, we compared the gut bacterial change after SG or RYGB at different levels. The composition of the phylum *Firmicutes* was decreased and the levels of *Bacteroidetes, Proteobacteria*, and *Actinobacteria* were increased after RYGB compared to sham operation (Figure 2A). However, SG did not induce a clear difference compared to SGS at the phylum level. At the genus level, SG and RYGB both induced *Escherichia* abundance (Figure 2B). However, a decrease in *Ruminococcaceae_unclassified, Lachnospiraceae_unclassified*, and *Ruminococcus* and an increase in *Porphyromonadaceae_unclassified, Bacteroides*, and *Akkermansia* were uniquely observed in rats after RYGB operation (Figure 2B). Moreover, SG and RYGB decreased the abundance of *Ruminococcus_*sp. and *Firmicutes_unclassified* (Figure 2C). However, an increase in *Alloprevotella_unclassified* was observed in both SG and RYGB (Figure 2C). Notably, the increase in *Desulfovibrionaceae_unclassified* and the decrease in *Oscillibacter_unclassified* and *Clostridiales_unclassified* were only observed in rats after RYGB (Figure 2C).

### SG and RYGB induced microbial function and metabolic pathway alteration

**Figure 3:**
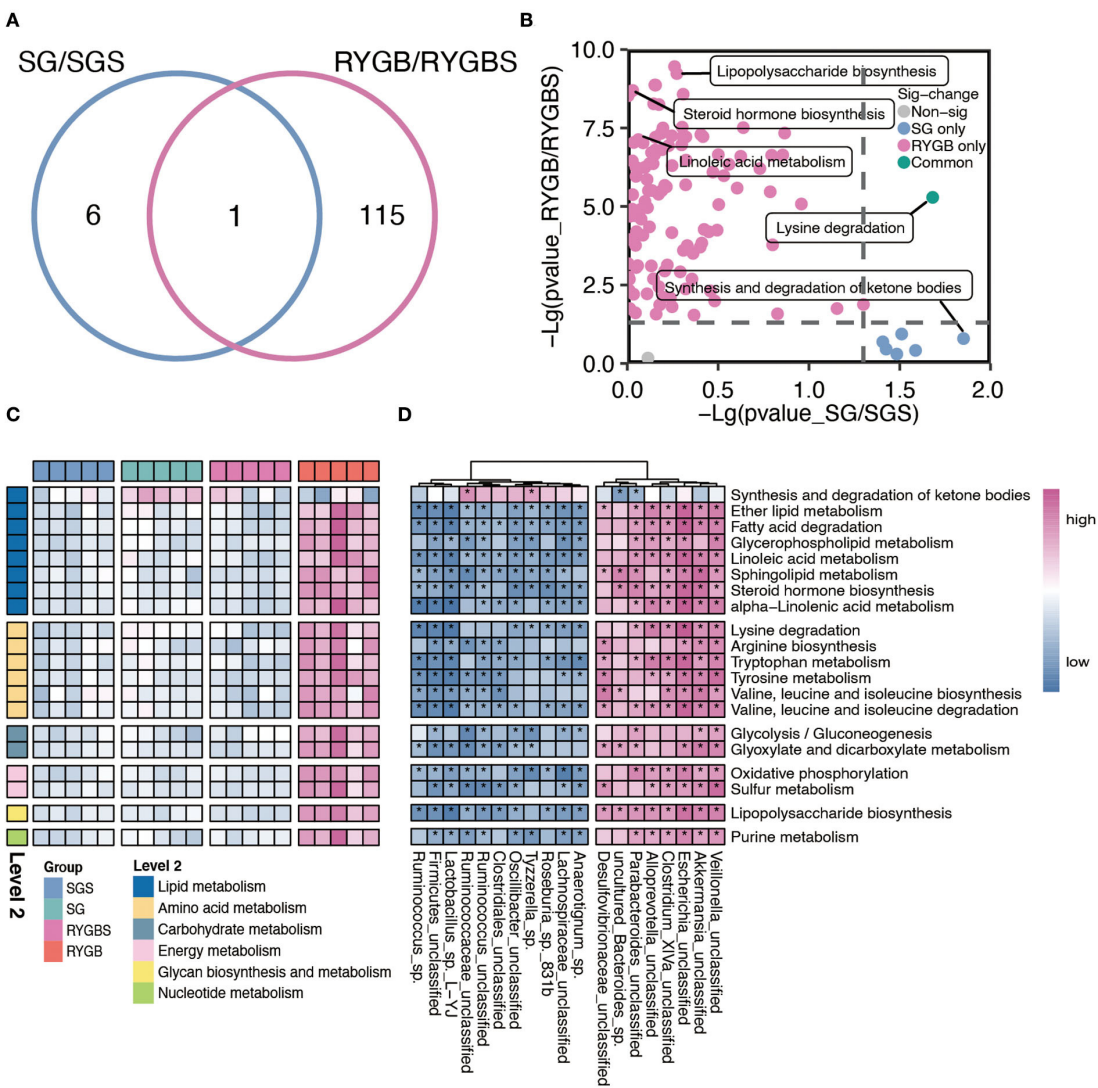
Characteristic function of gut microbiota in SG and RYGB rats. **(A)** A Venn diagram summarizing the differential and overlapping altered microbiota function between SG and RYGB relative to sham operations, respectively. **(B)** The clear but distinct difference between SG and RYGB compared to sham operations, respectively. **(C)** The predicted function of bacteria in the indicated groups. **(D)** The connection between the predicated function of intestinal microbiota and altered gut microbiota. Red denotes positive correlations. Blue denotes negative correlations. *P < 0.01.

Furthermore, compared to sham operation, both SG and RYGB showed a clear difference in the predicted function of gut microbiota. As shown in Figure 3A, only one pathway overlaps between the two comparisons. A large proportion of altered pathways were only induced by RYGB. Specifically, the RYGB uniquely enhanced the capability of lipopolysaccharide biosynthesis, steroid hormone biosynthesis, and linoleic acid metabolism, which belong to glycan biosynthesis, glycan metabolism, and lipid metabolism, respectively (Figures 3B,C). However, the increased ability to synthesize and degrade ketone bodies was only observed in rats after SG (Figures 3B,C). Importantly, both SG and RYGB could induce the alteration of lysine degradation (Figures 3B,C). In addition, the fatty acid degradation, ether lipid metabolism, BCAA biosynthesis and metabolism, purine metabolism, and glycolysis/gluconeogenesis were uniquely enhanced after RYGB (Figure 3C). In addition, we also observed a positive correlation between *Escherichia* and *Akkermansia* and lipids, amino acids, carbohydrates, energy and nucleotide metabolisms, glycan biosynthesis, and metabolisms (Figure 3D).

### Metabolomes revealed the RYGB-induced specific metabolic pathway change

**Figure 4:**
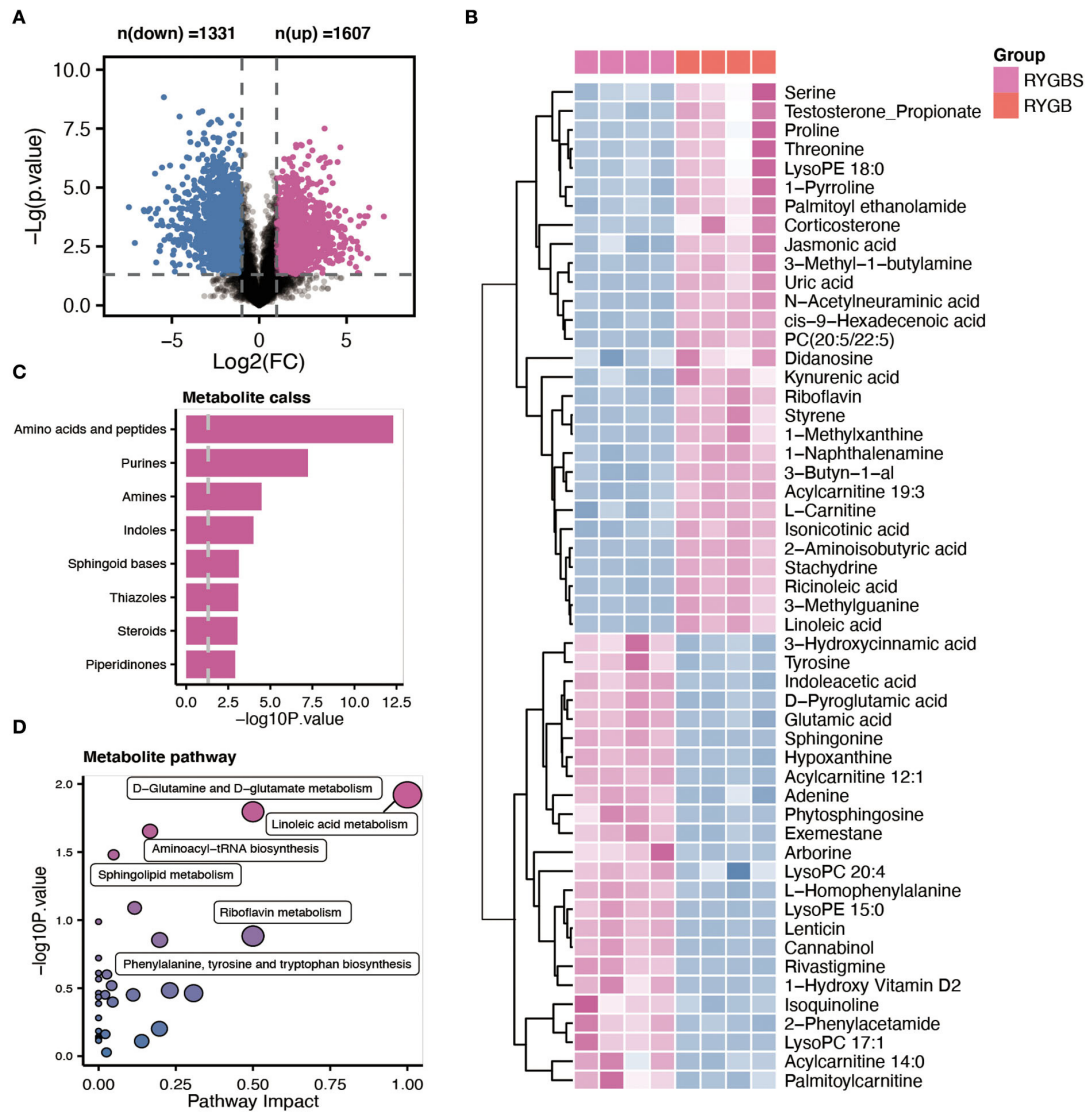
Comparative metabolomic analysis determines the change in plasma metabolites in RYGB rats from the sham operation. **(A)** The volcano plot shows the number of dysregulated metabolite features between the two groups. **(B)** The heatmap shows the annotated metabolites based on the KEGG database in the plasma profile. **(C)** Metabolic set enrichment analysis of different metabolites. **(D)** The KEGG pathway analysis based on the different metabolites.

Plasma samples from the samples were analyzed by the global metabolite panel, which identified a significant change in 1,331 (up) and 1,607 (down) features after RYGB operation (Figure 4A). As shown in Figure 4B, the abundance of serine, uric acid, PC (20:5/22:5), riboflavin, L–carnitine, ricinoleic acid, linoleic acid, and kynurenic acid was increased after RYGB. However, the downregulation of tyrosine, glutamic acid, sphingonine, adenine, LysoPC 20:4, and phytosphingosine was observed in rats after the RYGB operation (Figure 4B). Besides, the metabolic set analysis revealed that the changed metabolites are mainly enriched in amino acids and peptides, purines, amines, indoles, sphingoid bases, and steroids (Figure 4C). Furthermore, the KEGG pathway enrichment indicated that linoleic acid metabolism, D–glutamine, and D–glutamate metabolism, aminoacyl–tRNA biosynthesis, and sphingolipid metabolism pathways were remarkably altered after vitamin D treatment (Figure 4D).

### Integrated analysis of the gut microbiota and metabolism

**Figure 5:**
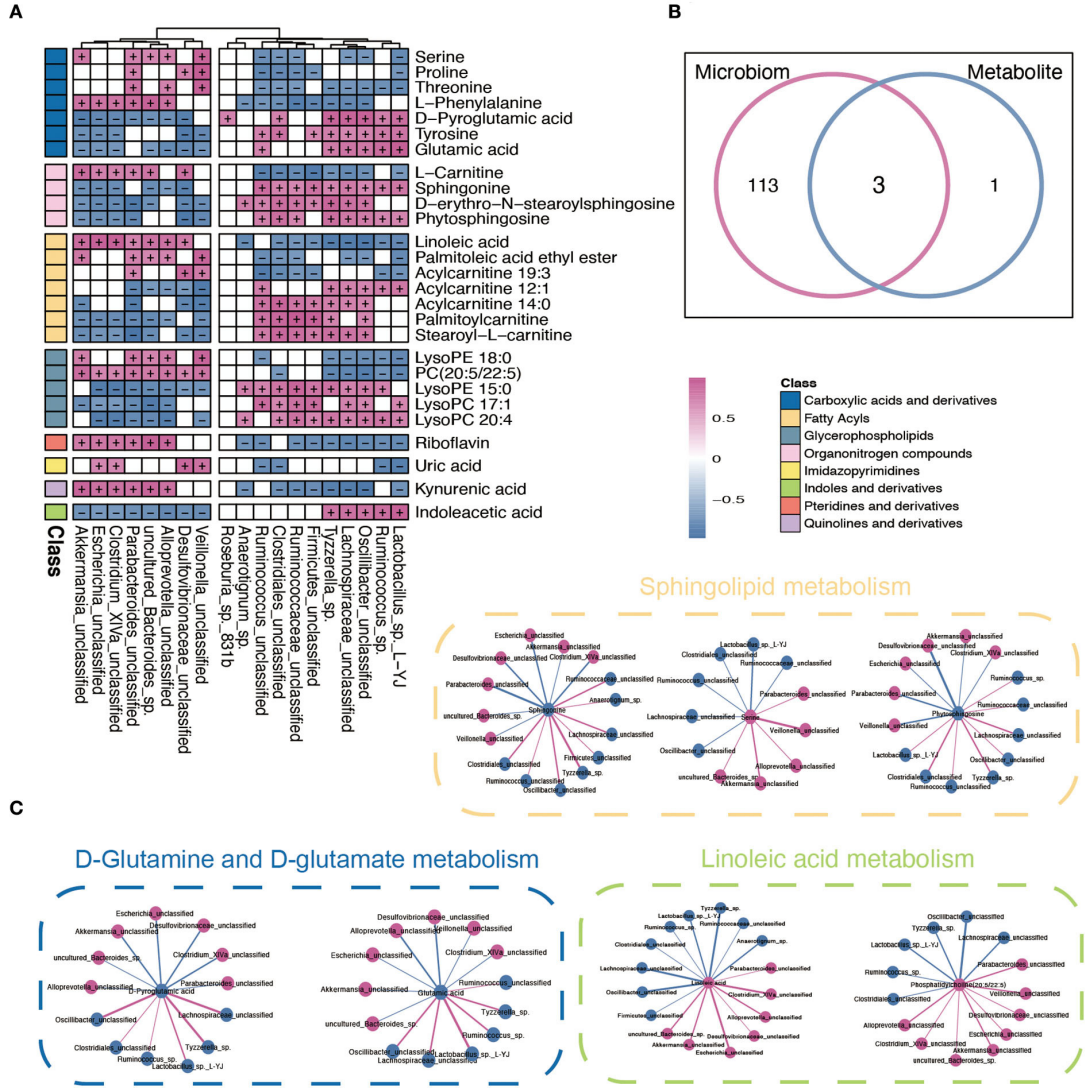
Integrated analysis between microbiome and metabolites. **(A)** The Spearman correlation analysis between significantly changed microbiome and metabolites. + and – indicate the significantly positive and negative association, respectively. **(B)** A Venn diagram of significant differential KEGG pathways in functional analysis of gut microbiota and the metabolic pathway based on the metabolites. **(C)** Interaction network plots of enriched metabolites in the shared pathways with microbes that participate in their metabolism. Red colors indicate significantly upregulated metabolites or microbes, while blue colors indicate significantly downregulated metabolites or microbes. Red and blue lines indicate the positive and negative correlation, respectively.

To further explore the potential influence of RYGB on both gut microbiota and plasma metabolites, Spearman's correlation analysis was performed to connect the changed metabolites and bacterial metabolites. As shown in Figure 5A, *Akkermansia_unclassified, Escherichia_unclassified, Clostridium_XlVa_unclassified*, and *Desulfovibrionaceae_unclassified* were positively correlated with L–phenylalanine, linoleic acid, PC (20:5/22:5), riboflavin, and kynurenic acid. However, the above metabolites were negatively associated with *Ruminococcus_sp., Oscillibacter_unclassified, Lactobacillus_sp._L–YJ*, and *Ruminococcaceae_unclassified* (Figure 5B). Importantly, the integrated analysis of significantly changed pathways revealed that the sphingolipid metabolism, D-glutamine and D-glutamate metabolism, and linoleic acid metabolism overlapped in the microbiome and metabolite (Figure 5B). Furthermore, the connection between the metabolites that belong to these metabolic pathways and altered gut bacteria is shown in Figure 5C. For example, *Parabacteroides_unclassified* and *Desulfovibrionaceae_unclassified* maintained a negative association with sphingonine and phytosphingosine. In addition, the linoleic acid and PC (20:5/22:5) strongly correlated with *Escherichia_unclassified* and *Akkermansia_unclassified*, respectively.

**Figure 6:**
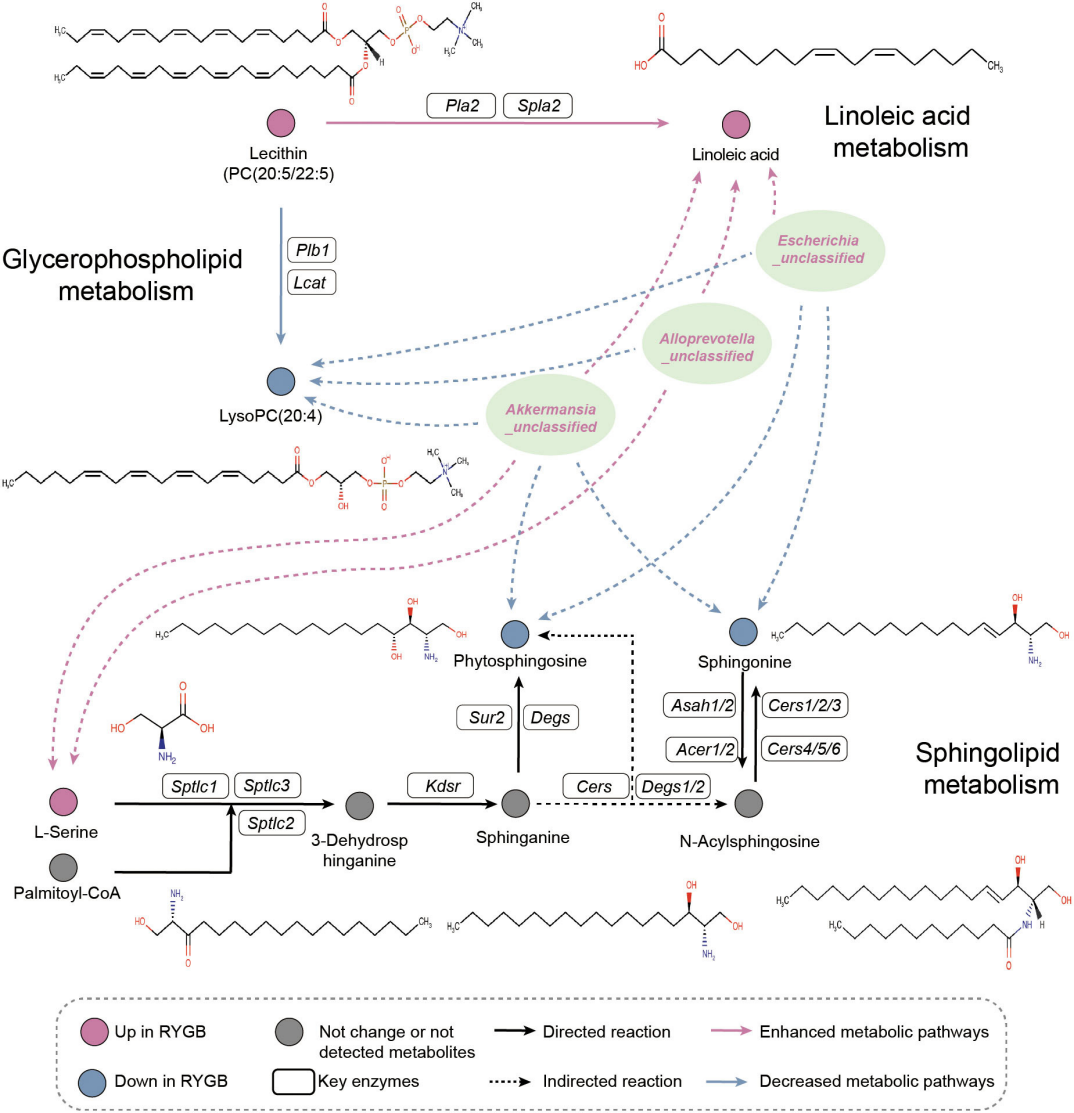
Pathway analysis. Illustrative examples of metabolite changes in glycerophospholipid metabolism, linoleic acid metabolism, and sphingolipid metabolism pathways and their correlation with the RYGB enriched gut microbiome.

As shown in Figure 6, the pathway analysis revealed that the ability of linoleic acid metabolism was enhanced from PC (20:5/22:5) after RYGB operation. However, the lysoPC (20:4) metabolism was inhibited from PC (20:5/22:5). Importantly, *Escherichia_unclassified, Alloprevotella_unclassified*, and *Akkermansia_unclassified* were found to be positively and negatively correlated with the plasma linoleic acid and lysoPC (20:4), which were produced from PC (20:5/22:5), respectively. Moreover, the increased abundance of *Escherichia_unclassified* and *Akkermansia_unclassified* after RYGB was negatively correlated with the phytophingosine and sphingonine metabolism from the sphinganine and N-acylsphingosine, respectively.

## Discussion

This study revealed that the two most common bariatric surgical interventions induced perturbative changes in microbial communities in rats. A previous study demonstrated that RYGB is more effective for inducing weight loss relative to SG (Ignat et al., 2017). Our study shows that RYGB induced more alterations in the gut microbiota compared to SG after the operation. The significantly downregulated F/B ratio in rats that underwent RYGB indicated the lower ability of calorie absorption *via* the gut microbiota. In addition, RYGB surgery induced the abundance of the phylum *Proteobacteria* and pro-inflammatory genus *Escherichia*, which is consistent with the previous cohort study (West et al., 2020). Besides, the increased levels of phyla *Verrucomicrobia* and genus *Akkermansia* indicated the absence of diabetes but the presence of obesity, liver steatosis, and inflammation (Cani et al., 2022). Furthermore, the higher level of phylum *Bacteroidetes*, including genera *Porphyromonadaceae* and *Bacteroides*, was reported to produce more butyrate and acetic acid to improve host metabolic function (Zhang et al., 2021; Tomioka et al., 2022). RYGB decreased the levels of genera *Ruminococcaceae, Ruminococcus*, and *Lachnospiraceae*, which have antioxidant capacity *via* the generation of reactive sulfur species (Uchiyama et al., 2022). RYGB surgery also decreased the abundance of fiber-degrading bacteria *Ruminococcus*, which was positively correlated with hepatic steatosis (Alferink et al., 2021; Turpin et al., 2022). *Alloprevotella* has anti-inflammation, balances blood glucose levels, and counteracts T2DM mice by producing short-chain fatty acids (Wei et al., 2018; Ge et al., 2022). *Oscillibacter* was linked to a decreased triglyceride concentration in plasma (Liu et al., 2022).

Consistently, a modest shift in gut microbiota function was observed in rats that received SG surgery compared to RYGB. The increased capability of ketone body synthesis in SG may reduce pro-inflammatory cells in the intestine as reported in a previous study *via* selectively inhibiting intestinal *Bifidobacteria* (Ang et al., 2020). Additionally, abnormal glycerophospholipid and sphingolipid metabolism were observed in the fatty liver (Kindt et al., 2018; Bauer et al., 2020). In addition, serum glycerophospholipids contribute to adipose tissue accumulation in obesity, which was positively correlated with metagenomic functional capacities for intestinal bacterial LPS synthesis and host markers of low-grade inflammation (Kayser et al., 2019; Wu et al., 2022). Therefore, the upregulated capability of glycerophospholipid metabolism indicates that the RYGB surgery may remodel the metabolic function by regulating gut microbiota.

Phosphatidylcholine was the most abundant phospholipid in all mammalian cell membranes involved in regulating lipid, lipoprotein, and whole-body energy metabolism and could reverse HFD-induced obesity, IR, and hyperglycemia (van der Veen et al., 2017; Gao et al., 2021). The linoleic acid supplement could reverse mucosal damage and inflammatory infiltrate in the colitis model, and the serum linoleic acid level negatively correlated with the odds of hepatic steatosis in a longitudinal analysis (Moreira et al., 2019; Makela et al., 2022). The negative association between *unclassified Escherichia, Alloprevotella*, and *Akkermansia* species and plasma lysoPC (20:4), as well as a strong correlation between these bacteria and linoleic acid, indicates that the RYGB may inhibit the production of linoleic acid and lysoPC (20:4) *via* PC (20:5/22:5) metabolism. In addition, the lower plasma carnitine decreased the capability of mitochondrial fatty acid oxidation, thereby contributing to the accumulation of free fatty acids in the liver (Zhao et al., 2020). Importantly, the enhanced level of plasma uric acid induced by RYGB may explain the higher risk of kidney disease accompanied by RYGB (Lieske, 2017). Taken together, the RYGB surgery produces a more robust and sustained fatty acid metabolic capability; however, the cost of more frequent side effects of RYGB should be considered.

Sphingolipids have been implicated in the development of a range of metabolic disorders, from insulin resistance (IR) to hepatic steatosis (Johnson et al., 2020). Sphingosine was closely associated with the inflammatory factor, and elevated sphingosine was also found in the blood of people with advanced diabetes (Cui et al., 2022; Sun et al., 2022). In addition, the decreased level of sphingosine implies the low activity of the rate-limiting enzyme *Cers*, which was positively correlated with IR (Johnson et al., 2020). However, phytosphingosine carry out anti-inflammatory activity *in vitro* and could decrease the inflammation in a mouse model of colitis (Montenegro-Burke et al., 2021). Thus, these findings indicate the potential role of RYGB in restoring systemic metabolic capability *via* modulating the sphingolipid metabolism.

Roux-en-Y gastric bypass induced the plasma L-phenylalanine, uric acid, and kynurenic acid, which were involved in metabolic syndrome (Li R. et al., 2021). Glutamic acid, one of the major excitatory neurotransmitters, is essential for the control of food consumption (Gong et al., 2020). Our results show a positive correlation between glutamic acid and *Oscillibacte*, which is consistent with the previous report (Liu et al., 2022). The RYGB surgery may inhibit the proliferation of *Oscillibacte* to decrease glutamic acid for regulating food consumption. However, whether *unclassified Escherichia, Alloprevotella*, and *Akkermansi* could affect linoleic acid, lysoPC (20:4), phytosphingosine, and sphingosine synthesis needs to be further confirmed. This study has several limitations. The male rats were adopted in our study. Further study of sex-dependent effects after weight loss surgeries should be considered. Even though the plasma metabolites in our study were a rich representation of systemic metabolic capability alteration, due to the active compounds of the gut microbiota-mediated metabolites, the fecal metabolome could contribute to a better understanding of the profound bacterial-metabolite crosstalk only after RYGB surgery besides the plasma metabolome. Although the altered gut microbiota were identified after RYGB, further study needs to be considered, such as verifying *via* single bacterial isolation or FMT.

## Conclusion

In summary, the SG surgery induced modest microbial alteration compared to RYGB. RYGB operation uniquely induced the abundance of unclassified *Escherichia, Alloprevotella*, and *Akkermansia*, as well as enhanced the capability of gut microbiota, glycerophospholipid metabolism, and sphingolipid metabolism. Plasma metabolism revealed an increase in PC (20:5/22:5), linoleic acid, carnitine, L-phenylalanine, uric acid, and kynurenic acid and a decrease in lysoPC (20:4), glutamic acid, phytosphingosine, and sphingosine after RYGB surgery. Of note, RYGB operation-induced *unclassified Escherichia, Alloprevotellad*, and *Akkermansia* were positively and negatively correlated with linoleic acid and lysoPC (20:4), phytosphingosine, and sphingonine, respectively. Taken together, this comprehensive analysis study revealed RYGB-induced perturbative changes in microbial communities and metabolic pathways provided there are clues of the RYGB in metabolic disease intervention *via* regulating fecal microbiome and circulating metabolism.

## Data availability statement

The data presented in the study are deposited in the GitHub repository, accession number https://github.com/123chenlei/RYGB.

## Ethics statement

All animal procedures used in this study were conducted according to the Guide for Care and Use of Laboratory Animals and approved by the Animal Ethics Committee of China Medical University (No. 2019099).

## Author contributions

JY (1st author) and JD designed and performed the experiments. LC and Y-LC conducted analyses and wrote the manuscript. X-YS contributed in data collection, fecal bacteria, and metabolite measurements. H-XL and JY (1st author) conceived and supervised the study. All authors read and approved the final version of the manuscript.

## Funding

This study was supported by grants from the Basic Research Project of the Educational Department of Liaoning Province [Grants JC2019022 and LJKZ0758].

## Conflict of interest

The authors declare that the research was conducted in the absence of any commercial or financial relationships that could be construed as a potential conflict of interest.

## Publisher's note

All claims expressed in this article are solely those of the authors and do not necessarily represent those of their affiliated organizations, or those of the publisher, the editors and the reviewers. Any product that may be evaluated in this article, or claim that may be made by its manufacturer, is not guaranteed or endorsed by the publisher.
